# Outbreak of human brucellosis in Southern Brazil and historical review of data from 2009 to 2018

**DOI:** 10.1371/journal.pntd.0006770

**Published:** 2018-09-18

**Authors:** Tamilly Silva Lemos, Juliana Clelia Cequinel, Tania Portela Costa, Amanda Boni Navarro, Andressa Sprada, Flávia Kazumi Shibata, Regina Gondolfo, Felipe Francisco Tuon

**Affiliations:** 1 School of Medicine, Pontifícia Universidade Católica do Paraná, Curitiba, PR, Brazil; 2 Secretaria de Estado da Saúde do Paraná, Curitiba, PR, Brazil; Oxford University Clinical Research Unit, VIET NAM

## Abstract

**Background:**

Human brucellosis (HB) is a bacterial zoonosis that is more frequent in low income and middle-income countries; it is sometimes associated with outbreaks. The aim of this study was to describe the largest outbreak of HB in Brazil.

**Methods:**

A retrospective cohort study of patients suspected of having contracted HB in the state of Paraná, Southern Brazil from January 2009 to January 2017. Following an outbreak of 51 cases of HB in a slaughterhouse at Paiçandu in 2014, HB was defined as an obligatory reportable disease in the State. Diagnostic tests for HB included serum agglutination, ELISA (IgG or IgM) and polymerase chain reaction (PCR). Clinical, laboratorial and epidemiological data were analyzed. A *P* value of 0.05 was considered statistically significant.

**Results:**

Out of a total of 3,941 patients, 754 presented with a positive test result for HB. After 2014, there was a significant increase in the number of cases, exceeding 100 cases per trimester. In the beginning of 2015, the workgroup of HB started several actions for prevention and treatment, and the number of cases progressively diminished to fewer than 20 cases per trimester. Of 191 reported cases, an occupational risk was found in 84.7%; most cases occurred in farmers (60.0%), veterinarians (17.6%) and slaughterhouse workers (14.7%). Manipulation of animals and unpasteurized milk consumption were associated with positive *Brucella* IgM ELISA with an odds ratio (OR) of 1.42 (1.09–1.84) and 1.48 (1.01–2.15), respectively.

**Conclusions:**

HB outbreaks can occur in low to middle-income countries and are associated with slaughterhouse work, handling of unpasteurized milk and animal manipulation. Intensive programs for control of HB are important to reduce the number of cases.

## Author summary

Human brucellosis (HB) is a bacterial zoonosis more frequent in low income and middle-income countries. The number of cases has increased in Southern Brazil since 2014. Considering the risk of dissemination of the disease, the authors evaluated the whole spectrum of the disease in the State of Paraná, where cases were reported. More than 3,500 patients at risk for the disease were evaluated and 754 presented with positive blood test results for human brucellosis. The local Health Agency established a program of brucellosis treatment and prevention in the following year. After the intensive control program, there was a significant reduction in the number of HB cases.

## Introduction

Human brucellosis (HB) is a bacterial zoonotic infection caused by *Brucella* spp. and is transmitted from several sources to humans. The main sources are cattle, sheep, goats, and pigs, which transmit the microorganism to humans through direct contact with infected animals or ingestion of contaminated food products [1]. The Gram-negative bacillus is transmitted through inhalation or the gastrointestinal route, causing a polymorphic acute or chronic inflammatory disease [2]. HB-related mortality rate was less than 1% of cases as reported by Buzgan et al. [3]. Nevertheless, the burden of disability caused by acute brucellosis is similar to that of acute malaria [4].

The burden of HB is not well defined because its incidence is always underestimated. Active surveillance of HB is not routinely performed, and most of the cases in low- and middle-income countries are poorly investigated [5]. The number of HB cases has decreased in industrialized countries, but it remains a concern in low- and middle-income countries.

In Brazil, brucellosis in cows [6], dogs [7], buffalos [8], sheep [9], goats [10], deer [11], horses [12], dolphin [13], and other animals has been reported. Human cases [14] and outbreak of laboratory-acquired *Brucella abortus* [15] have been described sporadically. However, serosurvey studies suggest that the infection is more prevalent than reported [16, 17].

Since the first case that was published in Brazil in 1934 [18], HB has been reported throughout the country, but it is generally restricted to workers of slaughterhouses, consumers of unpasteurized milk from areas of high incidence of bovine brucellosis [19], and agricultural workers [20]. In this study, we described the largest HB outbreak in Brazil.

## Methods

### Study design

We used the STROBE Statement for cohort studies to report the results and describe the methods. The study was approved by the ethical committee at PUC-PR (84644718.3.0000.0020). This retrospective cohort study included patients suspected to be infected with HB in the state of Paraná, Southern Brazil.

### Ethics statement

Informed consent was not necessary because this was a retrospective study. The authors guarantee the security of the data.

### Setting

In May 2015, HB was made statutorily reportable in Parana, Brazil. All probable or laboratory-confirmed new brucellosis cases were required to be reported. This decision was taken by the State Department of Health of Parana (SDHP) due to an outbreak comprising 51 HB cases in a slaughterhouse at Paiçandu in 2014. Thus, we evaluated the clinical data from January 2014 to January 2018 by active surveillance. Since March 2009, all laboratory tests for brucellosis are registered in the laboratory system of SDHP. We evaluated the positivity of serum tests for HB from March 2009 to January 2018. The tests used to detect HB included serum agglutination (Bengal Rose), ELISA (IgG or IgM), and polymerase chain reaction (PCR). When multiple tests provided different results in a patient, we considered only positive results. These results were used only for historical analysis of positive test results and not for case definition (see below).

### Case definition

Brucellosis cases were classified according to the guidelines for the management of HB in Paraná, Brazil [21].

*Suspected case*: any patient with acute or insidious fever plus clinical manifestations of HB plus an epidemiological link with infected animals or contaminated food or contact of a confirmed case.

*Confirmed case*: a suspected case with positive test results for *Brucella* spp. (serum IgM by enzyme-linked immunoassay [ELISA] or detection of *Brucella* DNA by PCR).

*Case excluded*: a suspected case with negative laboratory findings and/or a confirmed diagnosis for another disease.

### Laboratory tests

The current working group recommends laboratory tests for suspected cases and serology and molecular tests for the diagnosis of brucellosis. Laboratory tests required 2 mL of serum and 3–5 mL of blood to be collected in serum and ethylenediaminetetraacetic acid (EDTA) tubes, respectively. The serum was stored in a specific tube between 2°C and 8°C for 72 hours. After this period, the sample was stored at -20°C. The blood was then stored in EDTA tubes between 2°C and 8°C for 72 hours; the blood samples were not frozen. The materials used by this working group for the laboratory diagnosis of brucellosis were *Brucella* IgG and IgM ELISA (Serion, Maringa, Brazil) and real-time PCR. The Rose Bengal test (Laborclin, Pinhais, Brazil) has high sensitivity and specificity, but positive results can occur in asymptomatic patients after exposure to *Brucella* or after vaccination [22]. Real-time PCR is considered as the gold standard method for HB diagnosis because *Brucella* can only be cultured in laboratories with at least a biosafety level 3 [23]. The PCR test was performed using BD MAX, an open system with completely automated equipment, and the DNA-1 extraction kit and BD MAX DNA MMK with Sample Processing Control Master Mix (BD Diagnostic Systems, Québec, ON, Canada). Real-time PCR for detecting *Brucella* spp. was performed with primers and probes that targeted the bcsp31 gene (forward GCTCGGTTGCCAATATCAATGC and reverse GGGTAAAGCGTCGCCAGAAG) [24].

### Data source and variables

Data, including patient anamnesis, exposure, clinical manifestations, and results of *Brucella* laboratory tests (ELISA and PCR) were obtained by local physicians.

### Statistical methods

The incidence of HB was calculated based on the number of cases divided by the population of each city where cases were reported. The population sizes were obtained from the last population census conducted by the Brazilian Institute of Geography and Statistics in 2012 [25].

All variables were found to be correlated with the results of the serum *Brucella* IgG ELISA, *Brucella* IgM ELISA, and PCR. Moreover, a comparative analysis of all clinical and laboratorial data of confirmed and unconfirmed cases was performed.

Quantitative variables were expressed as mean with standard deviation (SD) and median with 25%-75% interquartile range (IQR). A comparative analysis of continuous data was performed using Mann-Whitney test (nonparametric) and Student’s *t*-test (parametric). Categorical data were analyzed using a chi-square or Fisher’s exact test (when any categorical data presented a value < 5 cases). A binary logistic regression was used to control confounding variables. A rate mapping was performed to visualize the change in the city over a period by city with free software TerraView (version 5.3.1). A *P* value of 0.05 was considered significant. For significant categorical data, the relative risk (RR) with a 95% of confidential interval (95% CI) was calculated. The statistical analysis was performed using SPSS 23.0.

## Results

**Fig 1:**
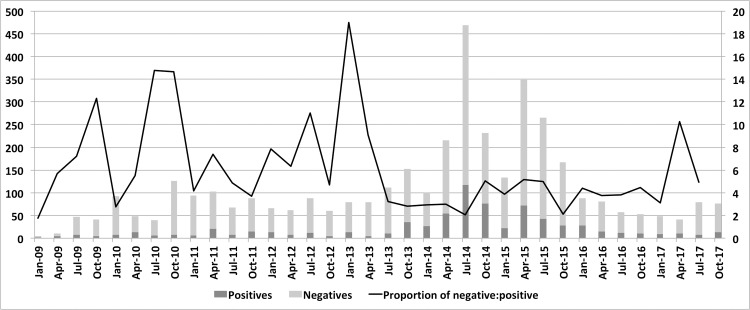
Number of positive (dark gray) and negative (light gray) test results for human brucellosis in the state of Paraná (Brazil). The line represents the proportion of negative test results for each positive test result.

A total of 9,523 tests for HB were performed in the state of Paraná between January 2009 and January 2018. However, within this period, 5,582 were repeated tests, and 3,941 were single tests (3,941 patients). From 3,941 patients, 754 tested positive for HB (673 by serum agglutination, 33 by ELISA IgG, and 48 by ELISA IgM). Fig 1 shows a histogram of the positive and negative test results in the period evaluated.

From 2009 to July 2014, only a small number of HB cases were reported (< 20 cases per trimester). However, after 2014, there was a significant increase in the number of cases, exceeding 100 cases per trimester. At the start of 2015, the HB workgroup started several preventive actions and treatments. After the guidelines were implemented, the number of cases progressively declined to fewer than 20 cases per trimester.

**Fig 2:**
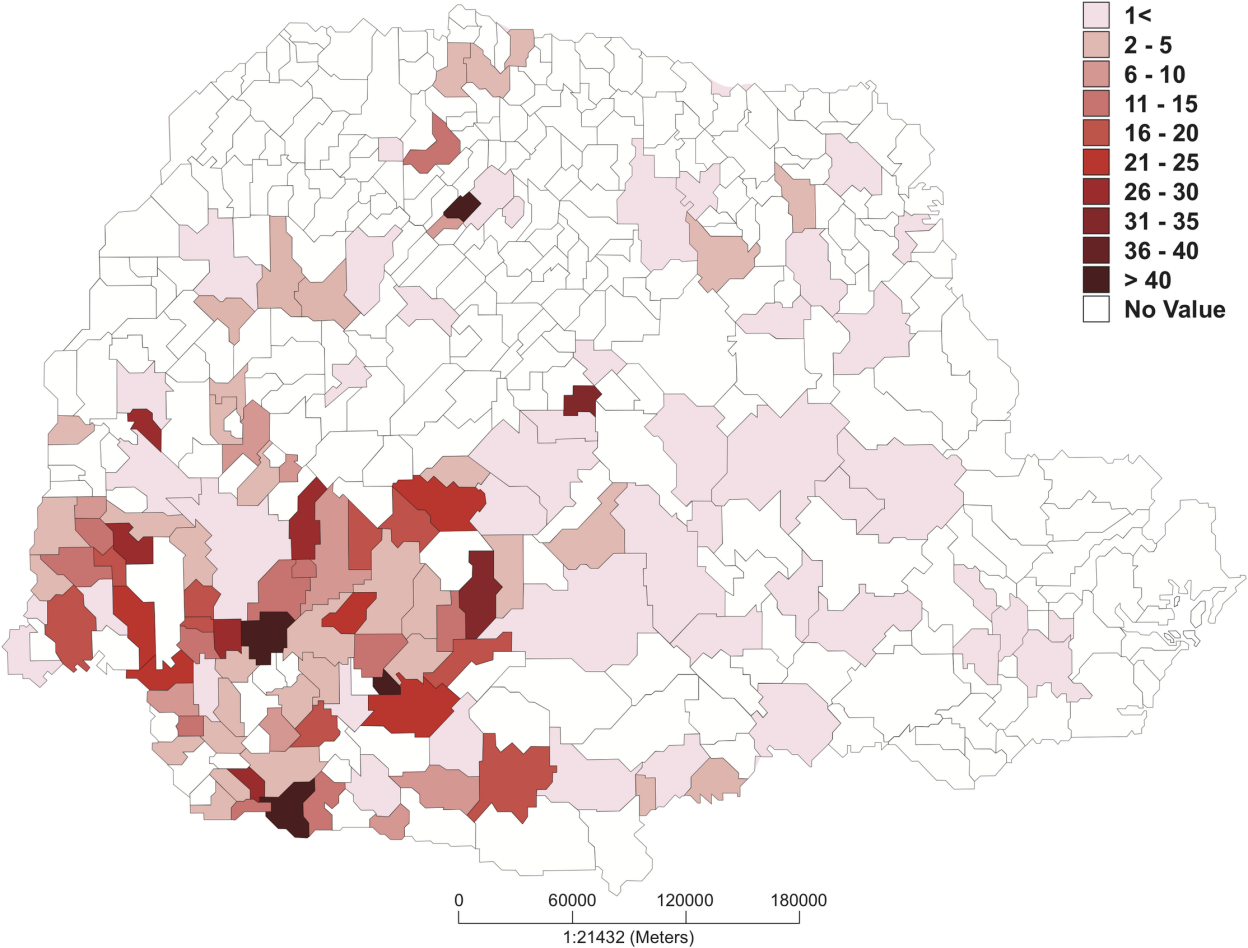
Map of the state of Parana and incidence of human brucellosis per city (cases/100,000 habitants/year) from January 2014 to January 2017.

Considering only the number of clinically available patients, a total of 191 suspected HB cases were reported: 55 (28.8%) cases in 2014, 47 (24.6%) in 2015, 23 (12.0%) in 2016 and 66 (34.6%) in 2017. Based on the number of cases in each year, the incidence of HB in the state of the Paraná was 0.49, 0.42, 0.20, and 0.59 cases per 100,000 population, in 2014, 2015, 2106 and 2017, respectively. The mean age was 37.7 ± 15.5 years (median 35, IQR 26–49) with higher prevalence in males (n = 126, 66%). Of 170 cases, 144 (84.7%) had occupational risk (21 had missing occupation data); most cases occurred in farmers (n = 102, 60.0%), veterinarians (n = 30, 17.6%), and slaughterhouse workers (n = 25, 14.7%). The state of Paraná is divided into 18 macroregions. Of the 18 regions, 9 accounted for 90.6% of cases (Fig 2). Approximately 74% of cases (n = 126) were from rural areas and 27.6% (n = 48) from urban areas. The distribution of cases per city is shown in Fig 2.

The prevalence of symptoms is detailed in Table 1. Myalgia/arthralgia (54.1%), fever (40.7%), and sweat (27.3%) were the most common symptoms. *Brucella* IgM ELISA was performed in 150 patients, and 89 patients tested positive for *Brucella* infection (59.3%). *Brucella* IgG ELISA was performed in 76 patients with 30 positive test results (39.5%). PCR test was performed in 59 patients, with amplification in 4 patients (6.8%). Based on the diagnostic criteria, 90 of 191 patients were diagnosed with confirmed HB (47.1%).

**Table 1:** Description of reported cases of HB, relationship of variables with the number of cases, and valid percentages based on the data obtained from January 2014 to December 2017.

Data		N	%
**Cases notified**		**191**	
**Confirmed cases**		**90**	**47.1**
**Sex**			
	Female	65	34.0
	Male	126	66.0
**Occupational risk**			** **
	Farmer	102	70.8
	Veterinarian	30	20.8
	Slaughterhouse worker	25	17.3
	Other	34	23.6
**Area**			
	Rural	126	74.0
	Urban	48	27.6
**Exposure and risk**			
	Animal manipulation	101	86.3
	Unpasteurized milk and derivatives	54	52.4
	RB51 vaccine accident	27	32.9
**Symptoms**			
	Myalgia/arthralgia	79	54.1
	Fever	55	40.7
	Sweat	35	27.3
	Weight loss	27	22.7
	Malaise/asthenia	42	32.6
	Headache	48	38.1
**Laboratory tests**			
	Positive IgM ELISA	89	59.3
	Positive IgG ELISA	30	39.5
	Amplification in PCR	4	6.8

All patients with suspect HB with positive *Brucella* IgG ELISA were analyzed to check correlation between the test and clinical findings. The clinical, epidemiological, and laboratorial data are shown in Table 2. Unpasteurized milk was the only factor associated with positive *Brucella* IgG ELISA. Similarly, all patients with positive *Brucella* IgM ELISA were analyzed to check for any correlation with a positive test. The clinical, epidemiological, and laboratorial data are presented in Table 3. Animal manipulation, unpasteurized milk, and weight loss were factors associated with positive *Brucella* IgM ELISA (OR = 1.42 (1.09–1.84), OR = 1.48 (1.01–2.15), and OR = 2.03 (0.92–4.33), respectively).

**Table 2:** Correlation of clinical, epidemiological, and laboratory data with positive *Brucella* IgG ELISA in 76 patients with suspected human brucellosis (HB).

		IgG -	IgG +	*P* value	OR (95% CI)
Data		(N = 46)	(N = 30)		
Epidemiologic					
	Male	30	18	0.412	
	Occupational risk	30	25	0.164	
	Rural area	32	14	0.098	
	Animal manipulation	23	10	0.477	
	Unpasteurized milk	25	5	0.010	6.00 (2.69–13.35)
	RB51 vaccine accident	5	3	0.141	
	Age (mean ±S D)	35.2 ± 17.8	36.8 ± 15.5	0.702	
Symptoms					
	Presence	34	20	0.483	
	Fever	19	18	0.444	
	Sweat	12	6	0.206	
	Myalgia/arthralgia	27	16	0.399	
	Weight loss	16	1	0.088	
	Malaise/asthenia	19	7	0.589	
	Headache	22	7	0.486	
Tests					
	Amplification in PCR	0	3	0.014	
	Confirmed HB	7	24	< 0.001	

**Table 3:** Correlation of clinical, epidemiological, and laboratory data with positive *Brucella* IgM ELISA in 150 patients with suspected human brucellosis (HB).

		IgM -	IgM +	*P* value	OR (95% CI)
Data		(N = 61)	(N = 89)		
Epidemiologic					
	Male	39	60	0.394	
	Occupational risk	39	74	0.069	
	Rural area	42	64	0.539	
	Animal manipulation	34	53	0.046	1.42 (1.09–1.84)
	Unpasteurized milk	23	20	0.023	1.48 (1.01–2.15)
	RB51 vaccine accident	2	7	0.551	
	Age (mean ± SD)	35.4 ± 15.8	40.3 ± 15.9	0.068	
Symptoms					
	Presence	37	41	0.107	
	Fever	21	19	0.333	
	Sweat	14	14	0.539	
	Myalgia/arthralgia	27	32	0.519	
	Weight loss	14	5	0.031	2.03 (0.92–4.43)
	Malaise/asthenia	17	13	0.235	
	Headache	21	15	0.168	
Tests					
	Amplification in PCR	1	1		
	Reagent IgG	7	21	0.007	
	Confirmed HB	1	86	< 0.001	

The 90 HB cases confirmed were found to be associated with animal manipulation, unpasteurized milk, exposure to RB51 vaccine, presence of symptoms, and weight loss (Table 4). A logistic regression was performed using the variables associated with confirmed HB cases, and none of the variables were independently associated with the confirmed HB cases.

**Table 4:** Correlation of clinical, epidemiological, and laboratory data with confirmed human brucellosis.

		Not confirmed	Confirmed	*P* value	OR (95% CI)
Data		(N = 98)	(N = 90)		
Epidemiologic					
	Male	65	61	0.365	
	Occupational risk	69	75	0.076	
	Rural area	62	64	0.124	
	Animal manipulation	34	53	0.037	1.49 (1.11–2.00)
	Unpasteurized milk	34	20	0.004	1.48 (1.01–2.15)
	RB51 vaccine accident	19	8	< 0.001	2.33 (1.27–4.28)
	Age (mean ± SD)	35.8 ± 15.2	37.7 ± 1 5.5	0.094	
Symptoms					
	Presence	60	43	0.040	1.38 (0.98–1.91)
	Fever	34	21	0.226	
	Sweat	20	15	0.497	
	Myalgia/arthralgia	44	35	0.472	
	Weight loss	21	6	0.029	2.00 (0.95–4.21)
	Malaise/asthenia	28	14	0.120	
	Headache	32	16	0.137	
Tests					
	Amplification in PCR	0	4	0.009	-
	Positive IgG ELISA	6	24	< 0.001	22.28 (6.69–74.22)

## Discussion

In Brazil, the first HB case was reported in Rio de Janeiro in 1934 [18]. Since then, the number of reported cases has increased. To our knowledge, this study is the first to report a cohort of cases after the implementation of compulsory reporting of the disease. This political decision was made after an outbreak of 8 confirmed HB cases in a slaughterhouse in Paiçandu (state of Paraná). The incidence of HB within this period was less than 1 case per 100,00 population. In China, reporting of HB cases has been compulsory for several decades. From 1970 to 2000, the incidence of HB was lower than 0.5 per 100,000 population [26]. In other endemic countries, such as Iraq [27], Azerbaijan [28], and Kyrgyzstan [29], the incidence reached more than 100 cases per 100,000 population. These low-income countries are now facing challenges in diagnosing HB, as it is often misdiagnosed as tuberculosis, Q fever, typhoid fever, and malaria [30]. The incidence in developed countries is extremely low and is more frequent in immigrant patients or occur in individuals after travelling [31].

In this study, the outbreak was clearly defined in 2014, with more than 100 cases per trimester and fewer than 20 cases per trimester in the previous years. After the outbreak in the slaughterhouse was identified, an educational approach was initiated in the specific slaughterhouse as well as in other cities, and cases declined progressively in the following year.

The HB we reported can be considered an occupational disease, as it is associated with exposure to contaminated animals in slaughterhouses and manipulation of animal products, including unpasteurized milk. Most of the patients were young male adults, had high occupational risks, and lived in rural areas. The map (Fig 2) showed an evident dissemination of the disease in the State of Paraná. Despite the intensive vaccination of cattle with RB51 as a matter of policy in the state of Paraná, the vaccine is only available for dairy cattle.

The frequency of HB symptoms is comparable with that reported in the literature. However, most cases were not adequately reported by the local physicians. This underestimated some symptoms and signs and it was impossible to classify the different forms of HB. Most cases were subjectively classified as acute cases with the following classical symptoms: fever, arthralgia, and weight loss. We identified certain chronic forms of HB, but most of them were not adequately classified.

The diagnosis of HB is a real challenge because cultures are not available. The state of Paraná does not have a safety level 3 laboratory, and previous diagnoses were based only on serum tests, specifically the Bengal rose test. After the outbreak in Paiçandu, a molecular test was standardized by the central laboratory of the state using *B*. *abortus* isolated from one patient as a positive control. Molecular techniques have been employed as important tests for several diseases; however, we cannot extend this concept for HB. In most cases with typical symptoms, positive ELISA serum test results and clinical response to HB therapy with aminoglycoside and doxycycline contrast with undetectable DNA by PCR. The positive control developed in the lab and used in the PCR had high accuracy, as reported in the literature, but we cannot extend these results to clinical practice [32–34]. Unfortunately, PCR was not performed in most cases due to inadequate blood samples. The protocol uses EDTA tubes, and only the tubes used for serology had been sampled. Most diagnoses were based on the symptoms and IgM ELISA, a well-established method in the literature, despite false positive test results [35–37]. Considering all the challenges to the diagnosis and management of HB, a study group developed local guidelines to help physicians, local epidemiology divisions, and veterinarians in the diagnosis, management after exposure, and reporting of the disease [21].

The correlation of serum tests with epidemiological data showed that positive *Brucella* IgG ELISA was associated with the consumption of unpasteurized milk. Positive *Brucella* IgM ELISA was also associated with animal manipulation. ELISA for *Brucella* IgG is a sensitive test for acute and chronic infections. Patients with past infections also present with a positive *Brucella* IgG ELISA, and in rare cases, this test can be falsely positive due to its sensitivity, which ranges from 90% to 100% [38, 39]. In the context of this outbreak, positive *Brucella* IgG and IgM ELISA correlated with the risk factors associated with HB. Serum tests were fundamental in the diagnosis of HB, and PCR presented a low positivity, but it was possible to diagnose one case based solely on PCR. Furthermore, PCR can be useful when serum tests are contradictory [40].

We describe the distribution of HB in the state of Paraná using the reported data and emphasize its recent reemergence. Improving our understanding of the epidemiology of this disease can help in the formulation of plans for regional and possibly national strategies to control HB.

## Supporting information

S1 Checklist Strobe checklist. (DOCX)

S1 Data Complete data. (XLSX)
